# Potentiality of LPS in Ameliorating the Histopathological Responses in Visceral Leishmaniasis‐Infected Mice

**DOI:** 10.1155/japr/8684617

**Published:** 2026-02-17

**Authors:** Ghusoon A. A. Al-Maphregy, Hazima mossa Alabassi, Ashwaq Ahmed Hussein

**Affiliations:** ^1^ Department of Biology, College of Education for Pure Sciences/Ibn Al-Haitham, University of Baghdad, Baghdad, Iraq, uobaghdad.edu.iq

**Keywords:** leishmaniasis, LPS, visceral leishmaniasis

## Abstract

**Objective:**

Visceral leishmaniasis is the second most fatal parasite illness worldwide, and it is the most severe type of leishmaniasis. LPS is a crucial chemical compound on the bacterial cell wall that the host recognizes and uses to launch an immune response to eliminate invasive infections.

**Materials and Methods:**

Four concentrations of LPS were used to treat infected mice with visceral leishmaniasis (20, 40, 60, and 80 ng/mL). Differential cell count and phagocytic index tests were done, then the liver and spleen were separated, and a histopathological assay was performed.

**Results:**

The findings demonstrated that all blood cells, with the exception of basophils, varied significantly between the uninfected and infected groups. With the exception of the 40 ng/mL concentration, there were notable variations in lymphocytes between the treated and infected groups as well as across the treated groups. With the exception of the 60 ng/mL concentration, neutrophils and monocytes showed significant changes between the treated and infected groups. Eosinophils, however, showed noticeable differences between the infected group and the treated groups. As for the phagocytic index, there was a significant difference between the uninfected and treated groups with the infected group. Histopathological results showed the effectiveness of LPS in the treatment of infected liver and spleen, especially in low concentrations (20, 40, and 60 ng/mL), whereas the highest concentration of LPS showed significant damage to liver and spleen tissue.

**Conclusion:**

This result opens up prospects for the possibility of using LPS as a treatment for many pathogens.

## 1. Introduction

Leishmaniasis, created by parasites spread through sand fly bites, is closely associated with poverty, disproportionately impacting individuals suffering from hunger, inadequate shelter, and homelessness [1]. The intramacrophage protozoa parasite of the genus *Leishmania* causes Leishmaniasis, a type of vector‐borne parasitic disease that is typically spread by at least 30 species of sand flies (of the *Phlebotomus* or *Lutzomyia* genera), though it is also occasionally spread congenitally and parenterally. Kala‐azar, another name for visceral leishmaniasis (VL), is diagnosed by fever, spleen and liver enlargement, weight loss, and the possibility of mortality if treatment is not taken [2]. The host–parasite connection is quite complicated in this systemic disease. Humans and a variety of mammals can serve as reservoirs for *Leishmania*, depending on the species [3].

Over 1 billion individuals are at risk of acquiring leishmaniasis because they reside in regions where the disease is endemic. Every year, an estimated 30,000 new cases of VL and over a million new cases of CL are reported [1]. One of the most significant endemic diseases in Iraq is leishmaniasis, which is primarily found in rural and peri‐urban areas, as well as in marshes and villages in the south of the country. In 1916, the first *Leishmania* infection was documented [4]. Histopathological alterations are linked to the infiltration of macrophages and lymphocytes carrying amastigotes in the liver and spleen, together with the hypertrophy and hyperplasia of the monocyte–mononuclear system, primarily in the liver and spleen [5]. The effective removal of these parasites from an infected host relies on the collaboration of various crucial components of the host immune system, which in turn influences susceptibility or resistance to infection by modifying the immune response [6].

In comparison to various other parasites, *Leishmania* employs an intracellular replication strategy to evade destruction by the host′s immune system; it effectively hides within a host cell and subsequently influences the development of lesions [7], particularly through its interactions with the innate components of the immune response.

Neutrophils and macrophages are the two most important phagocytotic cells at the bite site. Macrophages engulf the parasite once it is picked up by neutrophils [8]. The promastigote transforms into an amastigote inside the phagolysosome, which is more contagious and resistant to lysis than promastigotes, which are more susceptible to complement‐induced lysis. Uncontrolled replication and parasite spread within the human reticuloendothelial system, primarily in the VL, can occur when the parasite′s amastigote stage opposes the acidic environment inside the phagolysosome [9].

Notably, the parasite′s surface is coated in lipophosphoglycans, which stop the parasitophorous vacuole from attaching to the host cell′s lysosomes and creating hazardous complement complexes [10].

**Figure 1:**
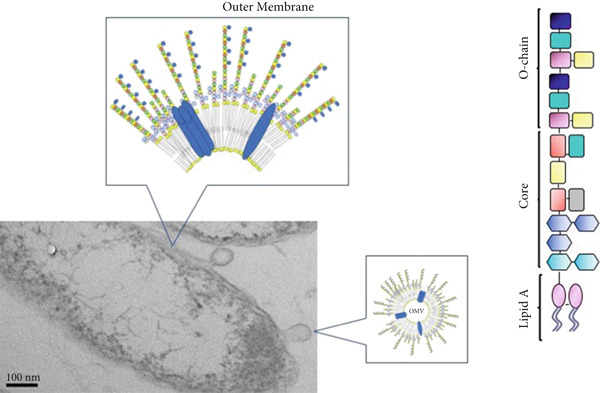
An illustration of the outer membrane of Gram‐negative bacteria, outer membrane vesicles (OMV), and an LPS molecular species, as well as electron microscopy of a *Ralstonia pickettii* bacterium.

The outer membrane of Gram‐negative bacteria is primarily composed of lipopolysaccharide (LPS). LPS is divided into three parts (Figure 1). The first of these potent compounds, lipid A, is a glycophospholipid moiety that anchors LPS in the bacterial membrane and is responsible for most of its biological effects. A core oligosaccharide frequently has lipid A moieties attached to it. Repetitive oligosaccharide units that extend outside of the bacteria make up the O‐chain, the third moiety of LPS molecules [11].

LPS, also called endotoxin, is well‐known to cause fever, systemic inflammation, and shock when injected intravenously [12]. Because of that, LPS has been recognized as a dangerous substance that should be eliminated in the medical field. Even with repeated low doses of LPS, systemic administration of LPS causes body weight loss in experimental animals [13]. For this reason, Mizobuchi et al. summarize previous studies on oral administration of lipopolysaccharides (OALs) and propose the potential of OAL as a new therapeutic strategy [14].

Even though LPS is a very inflammatory molecule, it has a wonderful therapeutic potential. Lipid A is the hydrophobic anchor of LPS and a glycolipid that binds strongly to the innate immune system through the Toll‐like receptor (TLR) 4/myeloid differentiation factor 2 receptor [15].

These molecules (LPS) target major physiological alterations and acute inflammatory reactions [16]. Thus, it should come as no surprise that LPS is regarded as an effective elicitor of the innate immune response and an early indicator of bacterial infections [17]. Because of its pro‐inflammatory qualities, it is frequently utilized to generate an acute–phase reaction in humans as well as animals [18]. Additionally, via binding to CD14 and the LPS‐binding protein, TNF‐*α*, IL‐1, and IL‐6 are among the inflammatory cytokines that are released when LPS triggers the inflammatory response [19, 20].

Increased pro‐inflammatory cytokines, MIF (migration inhibitory factor), and T‐cell activation are correlated with elevated LPS levels in VL, suggesting that this bacterial product may be associated with a compromise of immune effector function. LPS most likely originates from microbial translocation and may contribute to the immunopathogenesis of VL [21]. The complex viscerotropic *Leishmania*–host interaction′s immunopathogenesis is further complicated by LPS‐mediated cell activation. This immune activation can significantly impact the clinical course and prognosis of VL, increasing the risk of death even during antileishmanial treatment—a mechanism aggravated in *Leishmania*/HIV coinfected patients [22].

Interestingly, although LPS is a potent driver of inflammation, a growing body of research has also revealed its capacity to induce immunomodulatory and protective effects in certain contexts [14, 15]. Building on this paradoxical potential, this study is, to our knowledge, the first to investigate the therapeutic efficacy of LPS against VL. This novel approach is aimed at addressing the critical need for new treatments, as current antileishmanial therapies often involve lengthy hospital stays and painful injections of toxic drugs. The objective of this study is to explore the potential of LPS as a simpler, safer, and more accessible therapeutic strategy.

## 2. Materials and Methods

### 2.1. Preparation of LPS Concentration

A total of 10 mg of LPS (Medchemexpress, HY‐D1056) was dissolved in 100 mL of PBS (phosphate buffer saline) in order to make the stock solution: 10 mg/100 PBS = 0.1 mg (stock solution) and then four concentrations of LPS (20, 40, 60, and 80ng/mL) were prepared by using the formula below, and then they were stored until use [23]:

C1122 V=C V.C10.1= mg 100000 ng 11000000 mg= ng.V1100= ml PBS.C220406080=,, and ngmL.V2=?

### 2.2. Cytotoxicity Assay [MTT Assay]

The primary objective of this cytotoxicity assay (MTT assay) is to evaluate the potential toxic effects of LPS on normal human fibroblasts (NHFs). By assessing the viability of the cells in response to various concentrations of the test compound (LPS), the research is aimed at determining the compound′s cytotoxic profile. This will help us understand whether the LPS can cause cell death, which is crucial information for [safety testing, etc.]. Ultimately, the results of this test will contribute to achieving one of the research goals of testing the safe use of LPS in the oral cavity.

### 2.3. Cell Culture Conditions

MEM (US Biological, United States) was used to cultivate the NHF cell line. It was supplemented with 10% (v/v) fetal bovine serum (FBS) (Capricorn‐Scientific, Germany), 100 IU penicillin, and 100 *μ*g streptomycin (Capricorn‐Scientific, Germany). The culture was then incubated at 37°C in a humidified environment. Experiments were conducted using exponentially growing cells [24].

### 2.4. MTT Cytotoxicity Assay

A popular technique for assessing cell viability and cytotoxicity is the MTT (3‐(4, 5‐dimethylthiazol‐2‐yl)‐2, 5‐diphenyltetrazolium bromide) cytotoxicity test. The basis of this colorimetric assay is the capacity of living cells to use mitochondrial dehydrogenases to convert yellow MTT into purple formazan crystals. Cells are usually seeded in a 96‐well plate and exposed to several concentrations of the test substance in order to perform the MTT assay. Following the incubation period, MTT is added to every well and incubated once more. MTT is transformed by viable cells into formazan, which is soluble. A spectrophotometer is used to measure the absorbance at a certain wavelength, often at 570 nm, in order to quantify the concentration of formazan.

The quantity of viable cells directly correlates with the amount of formazan generated. Thus, cytotoxicity is indicated by a decrease in formazan formation (this means a decrease in absorbance) following LPS treatment [25].

### 2.5. MTT Procedure

In a 96‐well microplate (NEST Biotech, China), human fibroblast cell lines (NHF) were seeded at a density of 3 × 105 cells. They were then incubated for 72 h at 37°C until monolayer confluence was reached. By using the 3‐(4, 5‐dimethylthiazol‐2‐yl)‐2, 5‐diphenyltetrazolium bromide (MTT test) (Elabscience, China), cytotoxicity was examined. A range of concentrations (20, 40, 60, 80, and 100 ng/mL) was applied to the cells. Each well received 28 *μ*L of MTT dye solution (2 mg/mL) following a 72‐h exposure period. It was incubated for 3 h. Each well received 100 *μ*L of DMSO, which was then incubated for 15 min. A microplate reader was used to measure the optical density at 492 nm. The following formula was used to determine the percentage of cytotoxicity [26, 27]:

Cytotoxicity%=OD control−OD sampleOD Control×100.

OD control: mean optical density of untreated wells.

OD Sample: optical density of treated wells.

### 2.6. Culture of Parasite

Using Novy–MacNeal–Nicolle (NNN) medium and 100 IU/mL gentamycin, the *Leishmania donovani* strain (DUAA/IQ/2005/MRU15), which was kindly provided by the Baghdad University College of Science, was cultivated at 25°C–26°C [28].

### 2.7. Ethical Approval

This study was conducted at the Iraqi Center for Cancer Research and Medical Genetics at Al‐Mustansiriya University. In terms of scientific experiments involving animals, this center complies with the National Research Ethics Committees and the guidelines established under the 2018 Ethical Guidelines for the Use of Animals in Research, published by the National Committee for Research Ethics in Science and Technology (NENT). This institute was established in early 1998 with the goal of conducting state‐of‐the‐art scientific research on malignant diseases, including their causes, impacts, processes of occurrence, and treatment results.

### 2.8. In Vivo Study

The Cancer Research Center at Al‐Mustansiriya University provided 36 male Balb/c mice. All of the mice were 12 weeks old, weighed between 20 and 25 g, and were in good health. Following an intraperitoneal inoculation with 2 × 10^7^ promastigotes of *Leishmania donovani*, the mice were split into sex groups (each of which included six male mice) as follows:

1. The negative control group, or uninfected group (G1).
2. Infected but not treated group (G2) (positive control group)
3. Following that, four infected groups received various concentrations of LPS (G3) (20, 40, 60, and 80 ng/mL).

The treatment was given orally twice daily and for 1 month. At the end of the 4‐week treatment period, blood specimens were withdrawn from the understudied mice in order to perform the differential cell count and phagocytic index (PhI), then the mice were anesthetized using isoflurane (to exclude any harm that the mice may feel if the dissection is done without anesthesia). The mice were dissected after a few minutes, and the liver and spleen were separated for histopathological sections. The parasitic load was then quantified by counting the number of amastigote within macrophage per tissue section. The results were expressed as the number of amastigote per cell.

### 2.9. Histological Techniques [31]

These techniques include several stages, and they are as follows:

- *Fixation*: One of the first steps in getting specimens ready for microscopic inspection is fixation. Its goal is to keep cells and tissues in a “life‐like” state and stop deterioration. As soon as the organ was removed from the body, fixation began. Formaldehyde, also known as “formalin,” is the most widely used fixing agent. Prior to processing, the specimens were fixed by soaking in formalin for 12 h.
- *Processing*: We then allowed a succession of different solvents to penetrate the specimens, culminating in melted paraffin wax. The specimens first underwent a water‐based aquatic environment before being submerged in molten wax, which is hydrophobic and immiscible with water. They subsequently underwent multiple cycles of solvent dehydration and clearing, often using ethanol and xylene.
- *Embedding*: Following processing, the specimens were taken out of their cassettes and put in molds filled with wax at an embedding center. The specimen identifying details are contained in the cassette in which the tissue was processed. On top of the mold, it is now fastened with additional wax. The mold is removed once the specimen “block” has had time to solidify on a cool surface. Now a part of the block, the wax‐filled cassette provides a stable base for clamping in the microtome. The block containing the specimen is now ready for section cutting.
- *Sectioning*: The sections were cut using a microtome with very thin steel blades. Paraffin slices are usually cut to a thickness of 3–5 *μ*m. Before being placed on microscope slides, the sections were now “floated out” on the warm water surface in a flotation bath to flatten them. After they have dried, they are prepared for staining.
- *Staining*: The standard stain that is always used as a starting point for providing important structural information is hematoxylin and eosin (H&E) stain. In this stage, the cytoplasm and a number of extracellular components are dyed pink, whereas the cell nuclei are dyed blue. After staining, the pieces were covered with a glass coverslip.

### 2.10. Differential Cell Count

Using a Romanowsky stain, usually the Wright or May–Grunewald–Giemsa procedure, a drop of blood is thinly spread across a glass slide, left to air dry, and then stained to determine the differential blood count. The next step is to count and classify the 200 cells [29].

### 2.11. Fixation and Staining of Macrophages

Sterilized glass slides were smeared with peritoneal fluid and then incubated at 37°C. The phagocytic efficiency of control and hyperthyroid macrophages was examined by challenging them with eosin‐stained yeast suspension in vitro over a slide and incubating them at 37°C in a humid chamber for 3 h. Following incubation, three washes with 0.9% NaCl were used to get rid of the nonadherent cells. Methanol fixed the adhering macrophages. Giemsa was used to stain the macrophages after fixation, and they were then examined under a light microscope [30]. The following formula was used to determine the PhI:

Phagocytic index=Number of macrophages with phagocytosed yeast cellsTotal number of macrophages counted×100.

### 2.12. Statistical Analysis

To identify the significant differences between the groups under study, the SPSS software (V.28) was used to do a statistical analysis of the results using one‐way ANOVA with LSD. The findings were displayed as mean ± S.E., and a *p*‐value at *p* < 0.05 was deemed to indicate a significant difference between the groups′ means and between means parasites counts at *p* < 0.001 [32]. GraphPad Prism 8′s Tukey′s ANOVA multiple comparisons test was used to statistically assess the cytotoxicity assay data. The mean ± SD of triplicate measurements was used to display the values [33].

## 3. Results

### 3.1. The Effect of LPS on the NHF (Cytotoxicity Assay Results)

**Figure 2:**
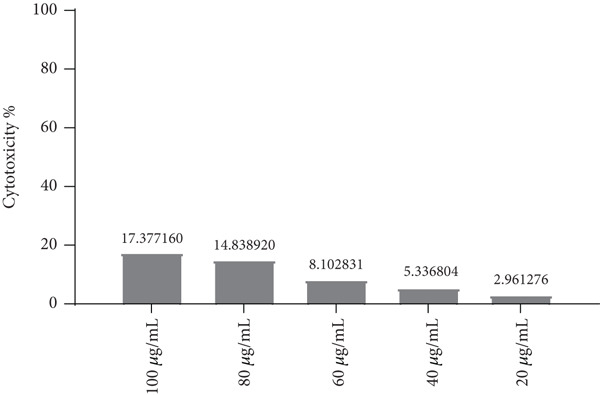
An inhibitory curve illustrating how LPS affects the viability of NHF cells. The LPS concentration is shown on the *x*‐axis, whereas the percentage of cell death (cytotoxicity) is shown on the *y*‐axis.

**Figure 3:**
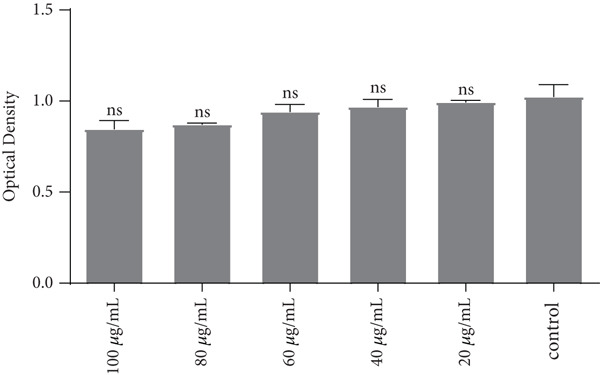
Reading curve for optical density (OD) for NHF cells exposed to LPS with time. LPS concentrations are shown on the *x*‐axis, whereas the OD value at 492 nm is shown on the *y*‐axis. The mean ± standard deviation of triplicates is represented by each data point.

According to statistical analysis of the outcomes of NHF treated with LPS, there were no appreciable changes between the LPS‐treated cells and the control cells after 72 h of incubation. At all used concentrations, the test compound did not cause cytotoxicity to cells (Figures 2 and 3).

**Figure 4:**
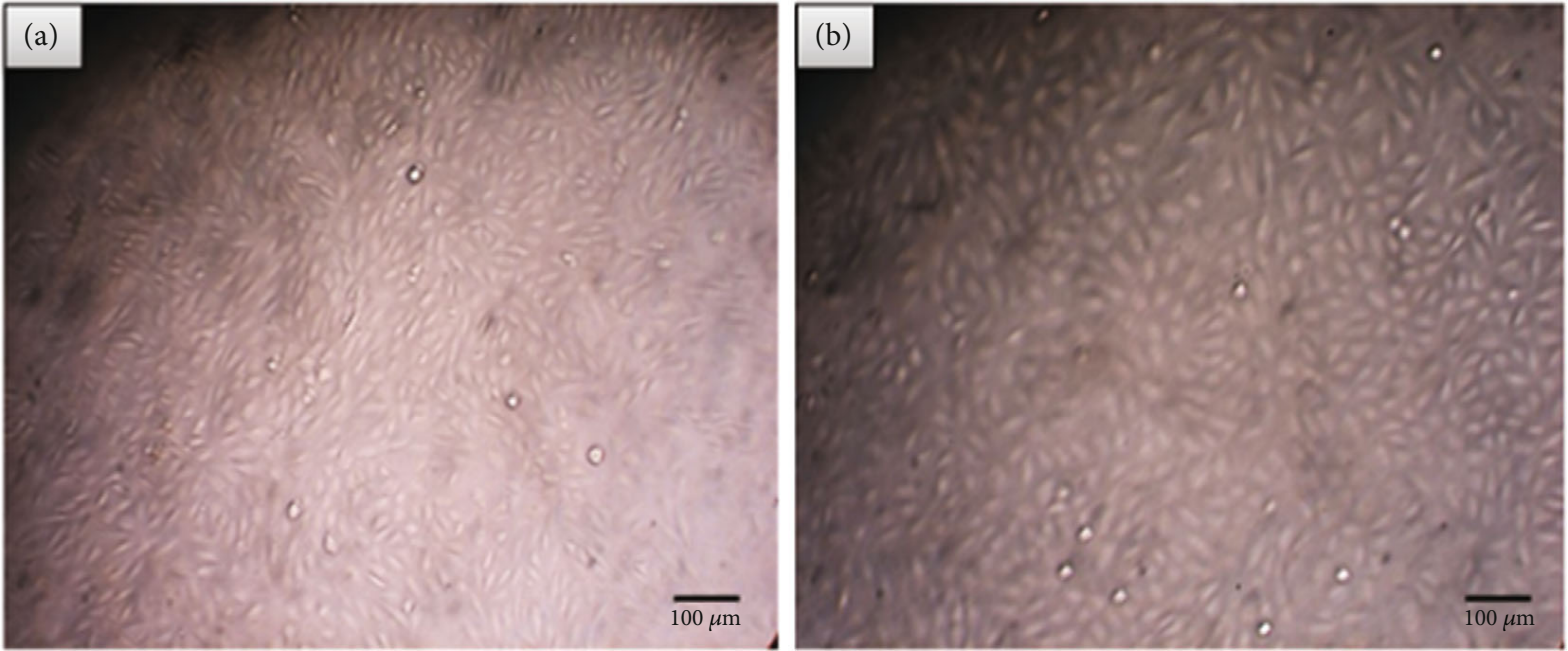
NHF before and after treatment with LPS. (a) untreated (control), (b) treated (sample) (X100).

Figure 4 shows that in terms of cell morphology analysis, the micrographs indicate that there were not any changes in cell morphology, such as cell shrinkage, membrane blebbing, or formation of apoptotic bodies, that could indicate cell death. Regarding cell density analysis, microscopic images indicate that there were no signs of cytotoxic effects, such as lower cell density compared with control wells. On the contrary, microscopic images indicate a high density of treated cells, which is similar to untreated (control) cells.

### 3.2. Histopathological Results

#### 3.2.1. Liver

##### 3.2.1.1. Normal Liver

**Figure 5:**
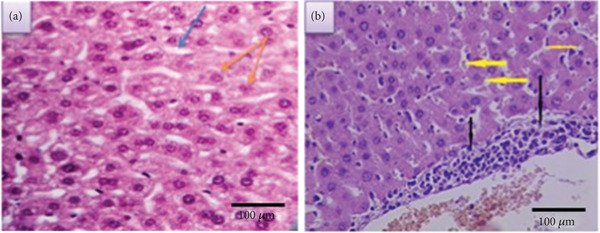
Section of (a) normal liver (blue arrow: sinusoid, orange arrow: hepatocyte) and (b) infected liver (black arrow: necrosis and inflammatory cell, yellow arrow: sinusoidal dilatation) (X400) (H&E).

The mouse liver has a lobular organization based on the distributions of portal regions and central venules, which is analogous to the livers of other mammalian species [34]. It is commonly known that the most important cells in the liver are hepatocytes, which make up 70%–80% of the liver′s cytoplasmic bulk. Vascular pathways known as sinusoids divide the hepatocytes, which are arranged in plates. The hepatic sinusoids include a type of cell called a Kupffer cell, which phagocytoses aged erythrocytes (Figure 5a).

##### 3.2.1.2. Infected Liver

VL, on the other hand, causes a focused necrotic region in the diseased liver section along with dilated hepatic sinusoids and the infiltration of inflammatory cells (mononuclear cells) (Figure 5b).

After mice were infected with VL, they were treated with different concentrations of LPS (20, 40, 60 and 80 ng/mL). Mice were dissected after 2 weeks and after 4 weeks. In both periods, liver and spleen were separated, to evaluate the effect of time and LPS dose size in treating affected tissues (liver and spleen).

##### 3.2.1.3. Treated Liver

**Figure 6:**
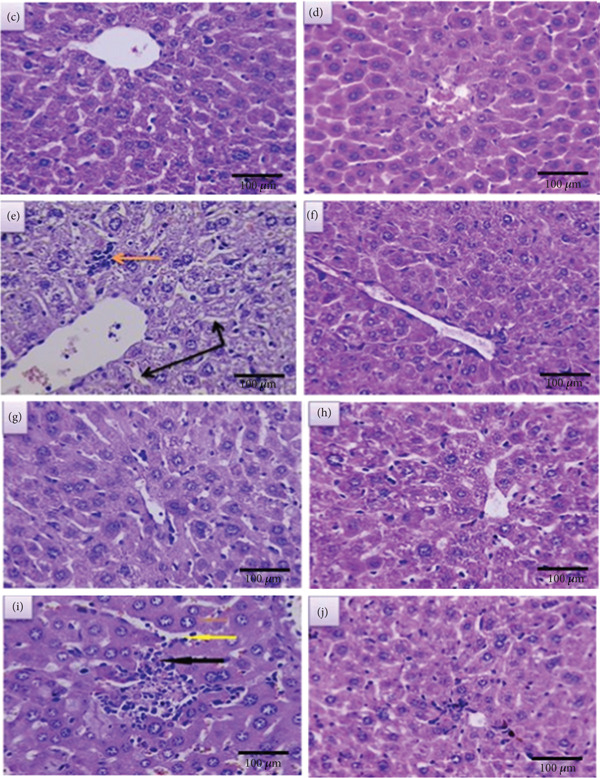
Sections of treated liver after 2 weeks (a, c, e, and g) and 4 weeks (b, d, f, h) (400X) (H&E).

*Treated liver With LPS After 2 Weeks* Figures 6a, 6c, 6e, and 6g represent sections of infected liver tissue treated with these concentrations of LPS (20, 40, 60, and 80 ng/mL, respectively). The first section shows near normal histological structure appearance with slight dilatation of sinusoid (Figure 6a), whereas the second section shows a slight depletion of sinusoid, focal area of necrosis and slight inflammatory cells infiltration (Figure 6c). The third section shows near normal histological structure appearance with a slight increment in Kupffer cells (Figure 6e). Finally, the fourth section shows a slight hypertrophy of hepatocyte, sinusoidal dilatation, a dispersed area of necrosis, and inflammatory cell infiltration (Figure 6g).

*Treated Liver With LPS After 4 Weeks.* Sections of infected liver tissue are shown in Figure 6b, 6d, 6f, and 6h, which were treated with these concentrations (20, 40, 60, and 80 ng/mL, respectively). The first section displays a very slight sinusoid dilatation along with an increase in Kupffer cells (Figure 6b). The appearance of the second and third sections resembles normal histological structure (Figures 6d and 6f). The fourth section displays hyperplasia of Kupffer cells, inflammatory cell infiltration, and a small focal region of necrosis (Figure 6h).

#### 3.2.2. Spleen

##### 3.2.2.1. Normal Spleen

**Figure 7:**
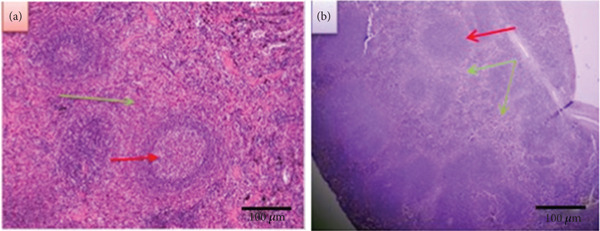
Sections of (a) normal spleen (red arrow: white pulp, green arrow: red pulp) and (b) infected spleen (red arrow: narrowing red pulp, green arrow: widening white pulp) (400X) (H&E).

The spleen in humans and mice is made up of red pulp with white pulp imbedded in it (Figure 7a).

##### 3.2.2.2. Infected Spleen

In contrast, the VL‐infected spleen section displayed narrowing of the red pulp and spreading of the white pulp (Figure 7b).

##### 3.2.2.3. Treated Spleen

###### 3.2.2.3.1. Treated Spleen With LPS After 2 Weeks

**Figure 8:**
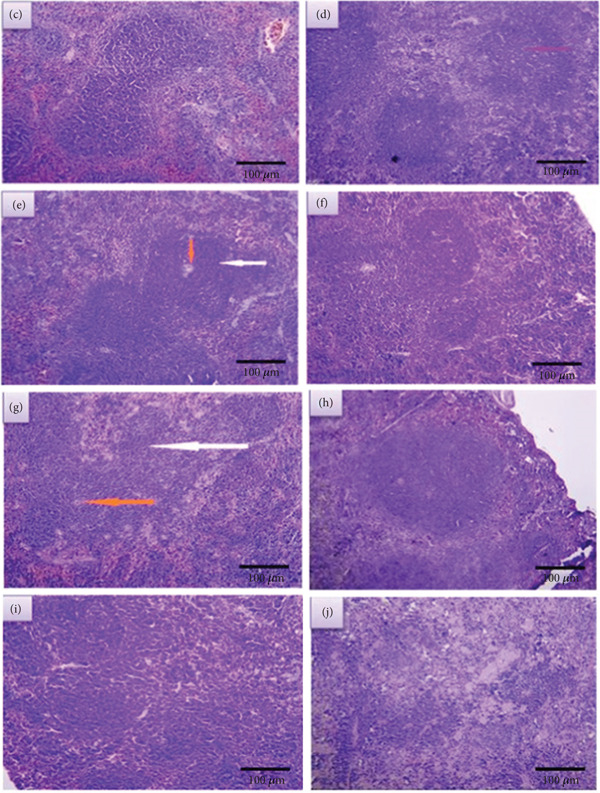
Sections of treated spleen after 2 weeks (a,c,e, and g) and 4 weeks (b,d,f, and h) (400X) (H&E).

Figures 8a, 8c, 8e, and 8g represent sections of infected spleen tissue that were treated with these concentrations (20, 40, 60, and 80 ng/mL, respectively.). The first section displays no red pulp and a broadening of white pulp (Figure 8a). In contrast, the second section exhibits a decrease in red pulp and an expansion of white pulp (Figure 8c). The third section shows prominent widening of white pulp (Figure 8e). Finally, the fourth section shows loss of histological architecture and depletion of lymphocyte cells (Figure 8g).

###### 3.2.2.3.2. Treated Spleen with LPS After 4 Weeks

Figures 8b, 8d, 8f, and 8h represent sections of infected spleen tissue that were treated with these concentrations (20, 40, 60, and 80 ng/mL, respectively). The first section shows a slight widening of white pulp with germinal center (Figure 8b), whereas the second, third, and fourth sections show widening of white pulp (Figures 8d, 8f, and 8h).

### 3.3. Quantification of Parasite Burden

**Figure 9:**
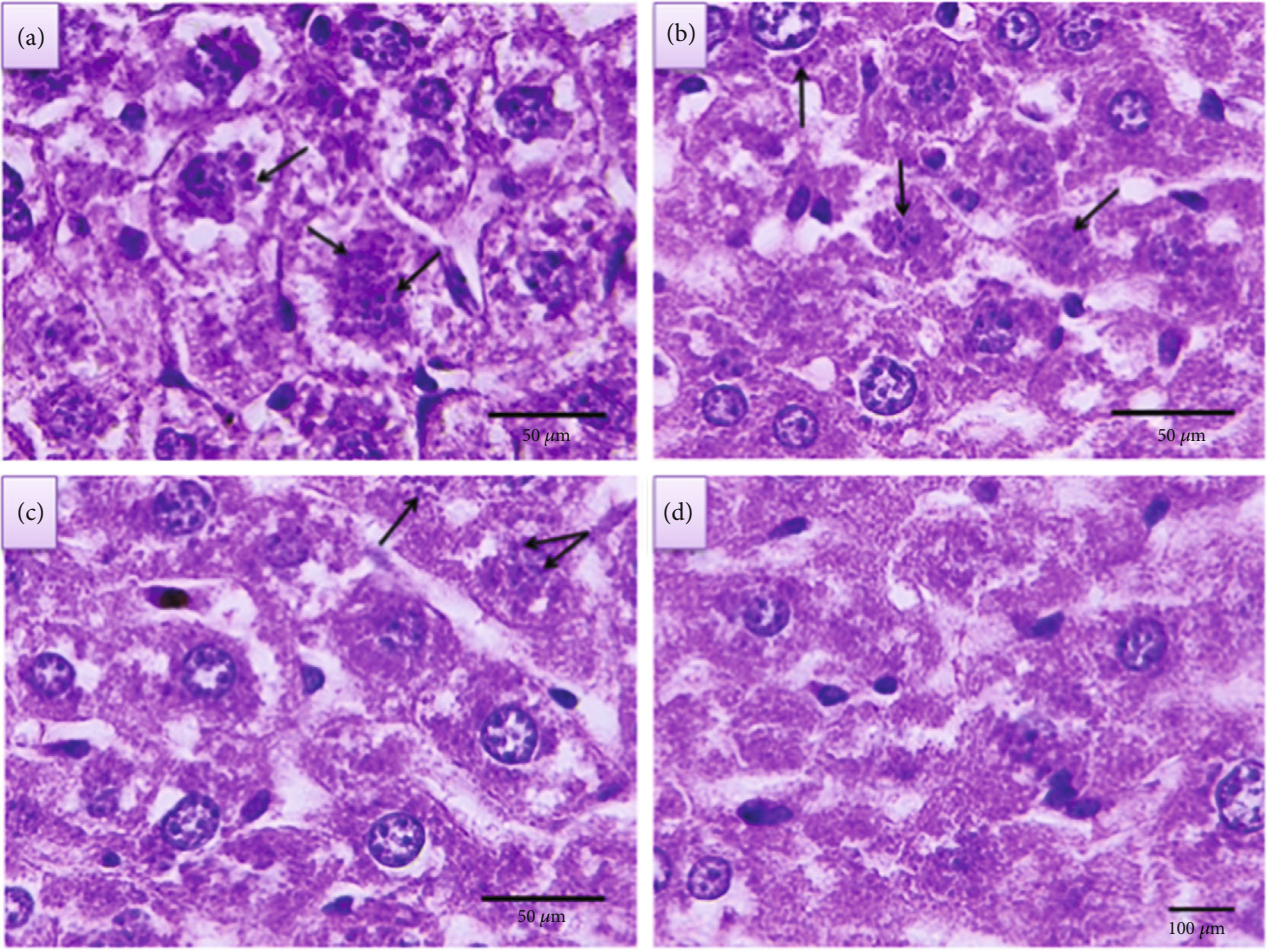
Histopathological sections of liver tissue after treatment with varying concentrations of LPS: (a) 20, (b) 40, (c) 60, and (d) 80 ng/mL (1000X) (H&E).

Following the treatment period, the parasitic burden in the liver was quantified by counting the number of amastigotes per macrophage in histological sections. The counts for each LPS concentration were averaged, and the results are presented in Figure 9 and Tables 1 and 2.

**Table 1:** Mean parasite count/cell following treatment with different LPS concentrations.

**Concentration (ng/mL)**	**Mean parasite count**
20	17.2
40	14.4
60	10
80	0

**Table 2:** Statistical comparison of mean parasite count across all LPS concentrations.

**Concentration (ng/mL)**	**Mean parasite count**	**p** **value**
20	17.2	< 0.001∗∗
40	14.4	
60	10	
80	0	

*Note:* The asterisks (∗∗) found inside Table 2 mean that there are highly significant differences.

Histopathological analysis of the liver sections (Figures 9a, 9b, 9c, and 9d revealed a dose‐dependent reduction in parasitic load. The number of observable amastigotes decreased as the LPS concentration increased. Notably, at the highest concentration of 80 ng/mL, parasites were completely eliminated from the observed tissue.

As shown in Tables 1 and 2, the mean parasite count decreased with increasing LPS concentration. Statistical analysis confirmed that these differences were highly significant (*p* < 0.001). Post hoc comparisons revealed that the 80 ng/mL concentration was the most effective, followed by 60 ng/mL. Furthermore, significant differences in efficacy were observed between the lower (20 and 40 ng/mL) and higher (60 and 80 ng/mL) concentration groups and between the higher concentrations themselves, but there were no significant differences between the lower concentrations themselves.

### 3.4. Immunoassay Results

#### 3.4.1. Differential Cell Count (Percentage)

**Figure 10:**
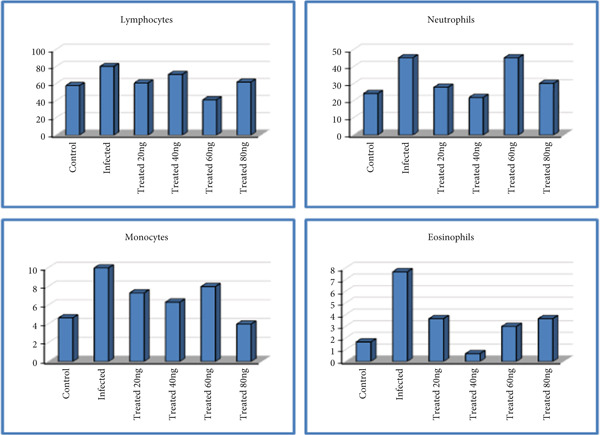
Comparison of blood cell count among all studied groups.

Tables 3 and 4 and Figure 10 show that there is a significant difference between the G1 and G2 groups for all the cells mentioned above except basophils. There were significant differences between the G3 and G2 groups for lymphocytes (*p* < 0.0001) except for the concentration of 40 ng, and significant differences between the G3 groupings themselves were also noted. Significant differences between the G3 and G2 groups were noted for neutrophils and monocytes (*p* < 0.002 and *p* < 0.019, respectively) except for the concentration of 60 ng. Also, significant differences between the G3 and G2 groups were noted for eosinophils (*p* < 0.003). As for basophils, these cells did not appear in any blood smear taken from the studied groups.

**Table 3:** The differential cell count of all studied groups.

**S**	**Replicates**	**Lymphocytes**	**Neutrophils**	**Monocytes**	**Eosinophils**	**Basophils**
1 Uninfected	A	54	25	6	2	0
	B	60	22	4	1	0
	C	60	26	4	2	0

2 Infected	A	78	40	8	9	0
	B	80	50	12	7	0
	C	83	46	10	7	0

3 Treated	A	57	33	7	3	0
	B	60	30	7	3	0
	C	66	21	8	5	0

4 Treated	A	76	16	7	1	0
	B	75	20	5	0	0
	C	62	30	7	1	0

5 Treated	A	41	35	12	5	0
	B	35	55	7	3	0
	C	48	46	5	1	0

6 Treated	A	58	32	3	7	0
	B	59	34	5	2	0
	C	69	25	4	2	0

**Table 4:** Comparison of blood cell count among all studied groups.

**Parameter**	**Groups**		**M** **e** **a** **n** + **S**.**E**	**p** **value**
Lymphocytes	Uninfected (G1)		58 ± 2	< 0.0001∗∗
	Infected (G2)		80.33 ± 1.45^a^	
	Treated groups (G3)	20 ng	61 ± 2.65^b,c^	
		40 ng	71 ± 4.51^a,c^	
		60 ng	41.33 ± 3.76^a,b,c^	
		80 ng	62 ± 3.51^b,c^	

Neutrophils	Uninfected (G1)		24.33 ± 1.20	0.002∗∗
	Infected (G2)		45.33 ± 2.91^a^	
	Treated groups (G3)	20 ng	28 ± 3.61^b^	
		40 ng	22 ± 4.16^b^	
		60 ng	45.33 ± 5.78^a^	
		80 ng	30.33 ± 2.73^b^	

Monocytes	Uninfected (G1)		4.67 ± 0.67	0.019∗
	Infected (G2)		10 ± 1.15^a^	
	Treated groups (G3)	20 ng	7.33 ± 0.33^b^	
		40 ng	6.33 ± 0.67^b^	
		60 ng	8 ± 2.08^a^	
		80 ng	4 ± 0.58^b^	

Eosinophils	Uninfected (G1)		1.67 ± 0.33	0.003∗∗
	Infected (G2)		7.67 ± 0.67^a^	
	Treated groups (G3)	20 ng	3.67 ± 0.67^b^	
		40 ng	0.67 ± 0.33^b^	
		60 ng	3 ± 1.15^b^	
		80 ng	3.67 ± 1.67^b^	

Basophils	Uninfected (G1)		0	Non
	Infected (G2)		0	
	Treated groups (G3)	20 ng	0	
		40 ng	0	
		60 ng	0	
		80 ng	0	

*Note:* A single asterisk means that there is a significant difference, whereas two asterisks mean that there are highly significant differences.

^a^Significant differences versus uninfected group.

^b^Significant differences versus infected group.

^c^Between treated groups.

### 3.5. PhI (Percentage)

**Figure 11:**
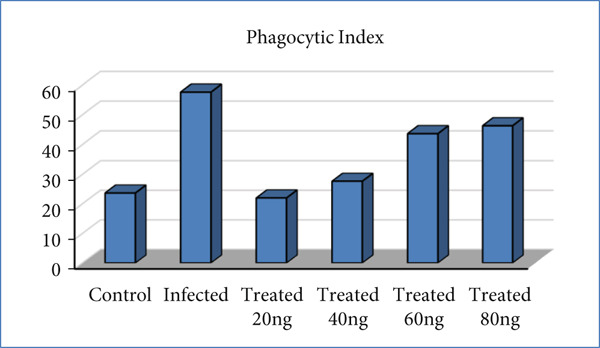
Comparison of phagocytic index among all studied groups.

Tables 5 and 6 and Figure 11 demonstrate that the G1 and G2 groups, as well as the G3 and G2 groups, differ significantly from one another (*p* < 0.0001). As for the treated groups, significant differences between concentrations (20 and 40 ng/mL) with the infected group were shown, whereas concentrations (60 and 80 ng/mL) showed significant differences with the normal and infected groups.

**Table 5:** Phagocytic index of all studied groups.

**S**	**Replicates**	**Phagocytic index**
1 Uninfected	A	19
	B	24
	C	28

2 Infected	A	60
	B	55
	C	58

3 Treated	A	20
	B	22
	C	24

4 Treated	A	27
	B	25
	C	31

5 Treated	A	40
	B	51
	C	40

6 Treated	A	45
	B	47
	C	47

**Table 6:** Comparison of phagocytic index among all studied groups.

**Parameter**	**Groups**		**M** **e** **a** **n** + **S**.**E**	**p** **value**
Phagocytic index	Uninfected		23.67 ± 2.60	< 0.0001∗∗
	Infected		57.67 ± 1.45^a^	
	Treated groups	20 ng	22 ± 1.15^b^	
		40 ng	27.67 ± 1.76^b^	
		60 ng	43.67 ± 3.67^a,b^	
		80 ng	46.33 ± 0.67^a,b^	

*Note:* Two asterisks mean that there are highly significant differences. The letter a means that there is significant differences between infected group (G2) and uninfected group (G1), also with treated group (G3), whereas letter b means that there is significant differences between the two concentrations (20 and 40 ng/mL) with the infected group only, whereas the other two concentrations (60 and 80 ng/mL) showed significant differences with the uninfected and infected groups.

## 4. Discussion

The primary characteristic of VL from an immunological view is its organ specificity. The liver and spleen are the primary responsive tissues that exhibit the pattern of immune response and parasite control, as demonstrated by studies on the progression of VL in mice. For this reason, the current study concentrated on these two organs

### 4.1. Liver

In regard to the liver infection with parasites, all the histological changes are considered an inflammatory reaction to injury induced by infection, but after treatment with LPS, we found a different pathological reaction that occurs in a dose‐ and time‐dependent manner.

Histological assessment indicated a significant improvement in liver tissue architecture at LPS concentrations of 20, 40, and 60 ng/mL, even in the absence of complete parasite clearance. The most pronounced tissue recovery was observed at 40 ng/mL. This dissociation between parasite burden and tissue pathology indicates that LPS confers a potent immunomodulatory benefit, potentially by modulating the host immune response to limit infection‐induced damage.

The results show that after 2 and 4 weeks with treatment of concentration of 20 ng/mL, the section appears with a slight dilatation of sinusoid, so the time has no impact on the final outcome of the injury‐induced wound. In regard to the concentration of 40 ng/mL, the result after 2 and 4 weeks of treatment revealed a different outcome. After 2 weeks, the infected mice liver showed a slight depletion of sinusoid, a focal area of necrosis, and slight inflammatory cell infiltration, while after 4 weeks, the liver showed a different result; it looked near a normal histological structure with a slight Kupffer cell infiltration. The third section of liver, which was treated with 60 ng/mL, also showed the same tissue change after 2 and 4 weeks, except for the disappearance of Kupffer cells after 4 weeks.

In contrast, a distinct outcome was observed at the 80 ng/mL concentration. Although this dose achieved complete parasite clearance, it induced significant histopathological damage in the liver. Analysis after 2 weeks of treatment revealed disseminated foci of necrosis accompanied by dense inflammatory cell infiltrates. However, by the 4‐week endpoint, the necrotic areas had reduced in size and were characterized by ongoing inflammatory cell infiltration and prominent Kupffer cell hyperplasia, indicating an active, but incomplete, tissue repair process.

### 4.2. Spleen

In the spleen section of infected mice without treatment, the result revealed that there was a widening white pulp; this reflects the inflammatory response. But in treated mice with 20 ng/mL after 2 weeks, the section revealed a different appearance. The white pulp has extreme widening with no red pulp, whereas after 4 weeks, there was a slight expansion of the white pulp with a germinal center. As for mice treated with 40 ng/mL of LPS, the section shows an increase in white pulp and a decrease in red pulp after 2 weeks. Additionally, the section shows an expansion in white pulp after 4 weeks. In regard to the 60 ng/mL after 2 weeks of treatment, the white pulp dilated and red pulp shrank in the spleen section; after 4 weeks, the section also showed dilatation of the white pulp. After 2 weeks of treatment with 80 ng/mL, the section shows a loss of histological architecture and depletion of lymphocytes, and after 4 weeks, there is a widening of white pulp.

So according to these results, it can be concluded that LPS works in a dose‐ and time‐dependent manner. Also, the immune stimulation caused by this substance seemed obvious through the expansion of the white pulp in most histological sections of the spleen

The histopathological damage observed in both the liver and spleen at the high LPS concentration (80 ng/mL), including lymphocyte depletion potentially due to apoptosis, indicates a dose‐dependent toxic effect in the murine model. This suggests that 80 ng/mL approaches the threshold of tolerability for mice. However, extrapolating this toxicity to humans requires careful consideration of species differences and administration route.

Notably, numerous studies have demonstrated that humans can tolerate significantly higher doses of LPS, particularly via oral administration. For instance, Kobayashi et al. [35] reported that oral LPS administration at doses of 0.01–1 mg/kg is effective in preventing and treating various diseases without reported toxicity. The safety of oral LPS is further supported by its natural presence in the human diet; edible plants such as wheat, rice, and buckwheat contain LPS, and daily oral intake has been associated with anti‐inflammatory, rather than pro‐inflammatory, effects [36, 37].. Furthermore, our in vitro cytotoxicity assay confirmed the safety profile of LPS for human cells, showing no toxic effects on NHFs at concentrations up to 100 ng/mL. Therefore, the tissue damage observed in mice at 80 ng/mL is likely a function of the high relative dose per body weight in a small animal, and not necessarily predictive of human toxicity, especially considering the proposed oral route of administration.

There were many factors that influence the immune response against *Leishmania* infection, such as the inoculation dose of parasite, which has an impact on inducing the inflammatory response in addition to the mouse′s genetic background [38]. The mechanism of the TLR signaling pathway and how LPS activates macrophages through TLR‐4 have both been extensively studied [39]. The two primary functional groups of macrophages are distinguished by their anti‐inflammatory (M2) and inflammatory (M1) characteristics [40]. Macrophage polarization to the M1 or M2 phenotype is induced by several activation techniques. In a study conducted by Khosrowpour et al. (2018) [41], in order to improve the immunopathological characteristics of inflammatory cytokines in infection, they discovered that *Leishmania* soluble antigen (LSA) can cause peritoneal macrophages to exhibit an immune‐regulatory phenotype. This study is different from our study; they link the immune‐regulatory effect to the LSA, whereas, in our study, this effect is linked to the immunostimulation by using LPS. Our study was in vivo, whereas Zahra′s study was in vitro, and we used *Leishmania* as a whole parasite, whereas Zahra′s study used the LSA, so there were many different points, but both results met at one point, the anti‐inflammatory and tissue‐reparative effects observed in our model, particularly at 40 ng/mL of LPS, can be interpreted in the context of dose‐dependent immunomodulation. The literature indicates that LPS dose significantly influence T cells polarization. Our finding that 40 ng/mL was the optimal dose for restoring liver architecture, despite persistent parasites, aligns with reports that low doses of LPS (e.g., <10 ng/mL) can induce a T‐helper type 2 (Th2) response [42]..We hypothesize that at this concentration, LPS may drive T‐cell differentiation toward a Th2 phenotype, characterized by the secretion of IL‐10 and TGF‐*β*. These cytokines can dampen excessive inflammatory responses, activate B cells for antibody production, and promote tissue repair, thereby explaining the observed histological improvement. Furthermore, the complex host–parasite–immune interaction may involve modifications to the innate immune recognition of LPS. As noted by Matsuura [43], the structure of lipid A, the core component of LPS detected by Toll‐like receptor‐4 (TLR4)/MD‐2, is critical. Certain pathogens can modify lipid A to a less‐acylated form, making it a poor stimulator of innate immunity.

It is speculated that the *Leishmania* infection might similarly alter the host′s response to exogenous LPS, potentially shifting the immune response toward a more regulated and less destructive profile. However, the exact nature of this interaction requires further investigation to elucidate the underlying mechanisms.

### 4.3. PhI

There was a highly significant increase in PhI in infected mice as compared with uninfected (*p* < 0.0001). The reason for this high increase, of course, is linked to the infection. Traditionally, parasite‐activated CD4+ T cells in infected mice divide quickly in the lymph nodes and release certain cytokines. Th1 secretes IL‐2, IFN‐*γ*, and TNF‐*α*, which activate macrophages and eradicate parasites [44]. But when comparing infected mice treated with 20 and 40 ng/mL of LPS for 4 weeks, we notice that the activation of phagocytes was decreased and the PhI decreased significantly as compared with infected mice (*p* < 0.0001). This result confirmed the pathological result for these two concentrations, especially 40 ng/mL, which returned the liver to its normal architecture approximately. This dose of LPS may drive Th2 polarization and activate the differentiation of T‐reg, which secrete the immune regulatory cytokines IL‐10 and TGF‐*β*. Also, this concentration may induce the differentiation of M2 and M*ϕ* but not M1 macrophages.

Treatment with high concentrations of LPS (60 and 80 ng/mL) for 4 weeks resulted in a significant reduction in parasite load, culminating in complete clearance at 80 ng/mL. Paradoxically, this potent antileishmanial effect was accompanied by a sharp increase in the phagocytic index compared with both infected controls and mice treated with lower LPS doses. We interpret this elevated phagocytic index not as a sign of enhanced immune function, but as a direct consequence of the histopathological damage observed in the liver and spleen at these concentrations. The tissue injury and inflammation likely stimulated a widespread, nonspecific phagocytic response by the reticuloendothelial system. The observed toxicity at 60 and 80 ng/mL is likely a species‐specific effect related to the high relative dose in mice. This is supported by safety studies demonstrating that LPS, when administered orally, exhibits remarkably low toxicity in other animals, even at doses of 500–4500 mg/kg, which are much higher than those that are toxic intravenously [45]. Therefore, the tissue damage in our murine model is probably a function of the animal′s small size and the administered concentration, and is not necessarily predictive of toxicity in larger species, including humans, especially via oral administration.

### 4.4. WBC Count

- Lymphocyte

The result showed a higher number of lymphocytes in concentration 40 ng/mL; this means that this dose is the best dose to stimulate the immune defense to treat the infection.

- Neutrophil

The higher result was within the concentration of 80 ng/mL, whereas the count of neutrophils was less than normal at concentration 40 ng/mL; also, this confirms the speculation that this dose is good for immunostimulation in order to treat the infection.

- Monocyte

There was a significant difference among all understudied groups. Classically, a monocyte will migrate to an organ and tissue to differentiate into a resident macrophage. The increasing count of monocytes may result as a consequence of cytokines releasing through the infection.

- Eosinophil

After 4 weeks, there was a significant increase in eosinophil in all treated mice (G3) as compared with infected mice (G2) (*p* < 0.003). The less count within a concentration of 40 ng/mL may indicate a therapeutic and tonic effect of the dose.

For decades, LPS has been utilized as a standard model for inducing sepsis and systemic inflammation via intravenous (IV) administration. Consequently, regulatory standards strictly limit endotoxin levels in injectable drugs, with the maximum tolerable IV dose in humans being as low as 4 ng/kg [46]. In contrast, a growing body of evidence, though less extensive than that for the IV model, indicates that OAL is associated with health maintenance and immunomodulation without systemic toxicity. For instance, OAL has been shown to controls granulopoiesis by promoting the commitment of hematopoietic stem and progenitor cells to the neutrophil lineage via a TLR‐4‐dependent mechanism [47]. Furthermore, lida et al. demonstrated that OAL can restore TNF expression and increase TNF‐producing leukocytes in the context of malignancies [48]. Critically, studies confirm the safety of this route; Soma et al. reported no toxicity when LPS was administered via oral or other mucosal membranes, affirming its potential to safety regulate innate immunity [49]. Additional research indicates that OAL can enhance macrophage phagocytic activity and exert neuroprotective effects by polarizing microglia toward a protective phenotype, all without inducing systemic inflammation [14]. Based on this compelling evidence for both safety and efficacy, oral administration was selected as the route for LPS delivery in our study.

## 5. Conclusion

This study establishes that LPS exerts a dose‐ and time‐dependent therapeutic effect against VL. A total of 40 ng/mL was identified as the optimal dose, effectively controlling the infection and facilitating tissue repair without the toxicity observed at higher concentrations (60 and 80 ng/mL). These findings position moderate‐dose LPS as a promising immunomodulatory strategy for VL.

## Conflicts of Interest

The authors declare no conflicts of interest.

## Funding

No funding was received for this manuscript.

## Data Availability

The data that support the findings of this study are available from the corresponding author upon reasonable request.
